# Challenges and opportunities in genetic improvement of local livestock breeds

**DOI:** 10.3389/fgene.2015.00033

**Published:** 2015-02-25

**Authors:** Filippo Biscarini, Ezequiel L. Nicolazzi, Alessandra Stella, Paul J. Boettcher, Gustavo Gandini

**Affiliations:** ^1^Parco Tecnologico Padano, Lodi, Italy; ^2^Institute of Agricultural Biology and Biotechnology, National Research Council, Milan, Italy; ^3^Animal Production and Health Division, Food and Agriculture Organization of the United Nations, Rome, Italy; ^4^Department of Veterinary Sciences and Public Health, University of Milan, Milan, Italy

**Keywords:** local breeds, genetic diversity, selection, genomics, phenotyping

## Abstract

Sufficient genetic variation in livestock populations is necessary both for adaptation to future changes in climate and consumer demand, and for continual genetic improvement of economically important traits. Unfortunately, the current trend is for reduced genetic variation, both within and across breeds. The latter occurs primarily through the loss of small, local breeds. Inferior production is a key driver for loss of small breeds, as they are replaced by high-output international transboundary breeds. Selection to improve productivity of small local breeds is therefore critical for their long term survival. The objective of this paper is to review the technology options available for the genetic improvement of small local breeds and discuss their feasibility. Most technologies have been developed for the high-input breeds and consequently are more favorably applied in that context. Nevertheless, their application in local breeds is not precluded and can yield significant benefits, especially when multiple technologies are applied in close collaboration with farmers and breeders. Breeding strategies that require cooperation and centralized decision-making, such as optimal contribution selection, may in fact be more easily implemented in small breeds.

## INTRODUCTION: THE FOCUS

Local breeds contribute across-breed genetic diversity to global animal genetic resources (AnGR). Unfortunately, many local breeds have a small population size which puts them at risk of extinction, according to the FAO (2013) system of categorization. Economics are a significant driver for loss of these breeds, as they tend to be less productive than common international transboundary breeds. Adding market value to local livestock breeds is a recognized strategy in conservation of AnGR (FAO, 2007), but the genetic improvement of breeds’ traits is also a concrete option for increasing their profitability. However, formal selection has rarely been implemented in local breeds. In this regard, we argue that the limited efforts observed are not due to the presence of insurmountable constraints. This paper reviews and discusses challenges and opportunities in implementing genetic improvement for local livestock breeds. We address our analysis to breeds categorized as being at risk because of their small population sizes.

## CHALLENGES

Although the constraints that breeds of small census size are faced with are not insurmountable, they are not trivial either. A first major challenge is simply a strong inertia built up by a typical small breed’s history; small breeds are usually not small by accident. Some breeds are small because they were developed and adapted to a given region and isolated by geographical constraints from expansion into wider markets. Others are small because of either deliberate policies to remain exclusive or a lack of effort or success in promotion and marketing. Others have never been as productive as the best breeds and were thus not competitive enough economically to appeal to a wide number of breeders. Small breeds suffer because they cannot take advantage of economies of scale in breeding and marketing programs. Larger breeds have a greater opportunity to increase selection response, because a larger number of individuals allows for greater selection differentials, especially when artificial insemination and other reproductive biotechnologies can increase the number of offspring per individual. Breeding companies also have more interest in larger breeds because the potential market is greater and because the truly superior animals are more extreme and thus more valuable. This vicious cycle basically condemns breeds to remain small under “standard” conditions. However, this reality does not preclude that particular interventions can be undertaken to overcome these obstacles.

### THE NEED FOR SELECTION PROGRAMS IN SMALL BREEDS

As explained, the inferior economic performance of a breed leads to decreased interest among farmers and eventually extinction. Therefore, some approach to selection is needed to increase economic performance: more output, less costs. In principle, all livestock breeds should be able to benefit from the advances in animal breeding and improvement. Obviously, differences exist across world regions and among breeds. For each breed, a critical first step is to undertake a SWOT (strengths, weaknesses, opportunities and threats; Martín-Collado et al., 2013) or similar analysis to identify logical breeding objectives and strategies to achieve those objectives. Some local breeds already have sufficiently high production to achieve profitability, but low performance with regard to secondary characters, such as with the udder conformation in the Reggiana cattle from Italy (Gandini et al., 2007). Other breeds may obtain greater benefits by improving output while maintaining their characteristic secondary traits, such as adaptation to the environment. For example, the Valdostana cattle are uniquely adapted to their mountainous production environment, but have relatively low milk yield. A well-designed selection program may seek to improve milk yield, but do this gradually to avoid creating energy demands that cannot be met by mountain pastures. At the same time, selection must avoid increasing body size and maintain leg conformation because these cows must go up to mountain pastures and negotiate steep slopes, meaning their center of gravity must remain low. In less economically developed countries, the importation of exotic breeds is often still proposed as a quick solution, but without adaptation failure is likely to occur, especially during years when climatic extremes occur. On the other hand, the time and capital invested in selection within breed will eventually lead to adapted and profitable populations (FAO, 2013). FAO (2013) has collected examples of successful selection programs in local breeds.

### GENETIC VARIATION WITHIN BREEDS

In conservation genetics, maintenance of both within-breed and across-breed genetic diversity are primary aims (Ollivier and Foulley, 2005), as they play different but critical roles in sustaining animal production. Selection can negatively affect both these components, and breeding programs should cautiously monitor genetic variation. Within a breed, the level and rate of inbreeding, which is negatively correlated with effective population size, is generally used as parameter of within breed variation. Index selection based on information from relatives leads to the reduction of effective population size and increases the probability of co-selecting close relatives (Wray and Thompson, 1990). This is particularly true for traits with low heritability. In designing selection programs for small populations, a main challenge is to maximize genetic gain at an acceptable inbreeding rate. Inbreeding rate per generation should be below 1.0% (Meuwissen and Woolliams, 1994), which may preclude selection when the population size is particularly small. However, as the population size increases, selection intensity can be increased, resulting in a continuum of situations with respect to selection differential. Different strategies are available to achieve genetic response with control of inbreeding, ranging from the earlier methods using sub-optimal criteria of selection (e.g., Grundy et al., 1994; Villanueva et al., 1994), to considering genetic relationships among selected animals (e.g., Brisbane and Gibson, 1995), to the most-sophisticated selection with optimal contributions (OC; Meuwissen, 1997; Grundy et al., 1998).

Various studies have compared OC selection retrospectively with the observed values after truncation selection. Potential for up to 30% more genetic gain at a given inbreeding rate was revealed in Meatlinc sheep and Aberdeen Angus populations (Avendaño et al., 2003). Koenig and Simianer (2006) observed 13% more genetic gain from OC selection compared to the actual breeding program for German Holstein bulls, under the same average relationship constraint. These studies determined that a lack of inbreeding control had resulted in unbalanced use of ancestors in these populations and underline the benefits of OC selection. This is particularly true in local breeds with limited population size, where inbreeding rates are expected to be high using conventional selection.

When acceptable rates of inbreeding are not known *a priori*, it is possible to generate a response surface of inbreeding versus genetic gain to facilitate the choice of the inbreeding level to be adopted (Brisbane and Gibson, 1995; Meuwissen, 1997).

### GENETIC VARIATION AMONG BREEDS

The genetic diversity of a livestock species is generally addressed by keeping a sufficient number of breeds. However, species-wide diversity must also be considered during selection within a breed. In general, FAO (2013) suggests that selection should conserve breeds as genetically and cultural distinct genetic resources. Selection for increased output while ignoring traits correlated to traits of conservation interest such as adaptation, specific genetic variants, and quality of products, can reduce breed distinctiveness and between-breed variation. Identification of selection traits in local breeds should be accurate and based upon knowledge of the trait biology. Advances in genomics and bioinformatics have allowed the identification of genomic similarities/differences among livestock breeds (see de Simoni Gouveia et al., 2014 for a review). Some of these genomic signatures may contribute to explain the phenotypic uniqueness of breeds (Huson et al., 2014; Somavilla et al., 2014) and facilitate prioritization and the use of genomic breeding tools to preserve these important traits. A further option is the landscape genomics approach, whereby the association between alleles and geographic locations and/or climatic variables is targeted and assumed to be suggestive of signatures of adaptation, giving information on the environmental forces acting on the genome (Joost et al., 2013).

### ECONOMIC SUSTAINABILITY

Genetic improvement programs require significant investments. Although well-designed genetic improvement can be expected to eventually provide positive returns on investments, in local breeds the costs will often be relatively high on a per-animal basis. The breeding strategy and system that maximize genetic response may not be optimal from an economic standpoint. Recording of performance and pedigrees may not be economically sustainable, even if restricted to a portion of the population (e.g., the nucleus). Lack of infrastructure in marginal areas where local breeds are often found may impair the introduction of genetic improvement programs, and the development of the infrastructure may be costly. When considering artificial insemination, the production of few semen doses per donor, as expected in small populations, can substantially increase per-dose semen costs relative to large populations. Therefore, a cost–benefit analyses should be conducted before implementing selection in local breeds to determine the optimal approach. In genetic improvement programs, economic returns should be evaluated in the long term, given generation intervals and genetic cumulative effects. Additionally, organizational and infrastructural shortcomings are often associated to local breeds: these could be circumvented by taking advantage of existing organizations and infrastructures developed by larger breeding organizations: examples are the genetic evaluation for Meuse–Rhine–Yssel cattle carried out by CRV^1^ in the Netherlands, and regional breeds cattle data managed by ICBF^2^ in Ireland.

## OPPORTUNITIES

### GENOMICS

In the last decade, low- and high-density genomic tools (Lenstra et al., 2012; Utsunomiya et al., 2014) have been broadly used to study and characterize the genetic diversity and population structure of livestock (FAO, 2011; Rothschild and Plastow, 2014). However, genomic information has contributions beyond characterization to make to the management and conservation of AnGR. In addition to identifying genomic regions subject to natural selection, signatures of artificial selection have been identified applying statistical approaches to genomic data (Stella et al., 2010; Randhawa et al., 2014). Other uses of genomic information with potentially high impacts on management include: the estimation of genome-based relationships and inbreeding coefficients, from single nucleotide polymorphisms (SNPs; e.g., Manichaikul et al., 2010; VanRaden et al., 2011a), from runs of homozygosity (e.g., Purfield et al., 2012), or unifying different sources of information (e.g., Wang and Da, 2014); genetic approaches for breed-based product identification and traceability, for authentication and quality assurance (Nicoloso et al., 2013); and the identification of recessive lethals or other specific mutations of interest (VanRaden et al., 2011b; Pirola et al., 2013). All of the above can be applied to small breeds at relatively low cost (e.g., using low-density or custom panels). However, as is the case for the analysis of any breed that had not been considered in the development of the SNP chips, ascertainment bias should be carefully taken into account (Albrechtsen et al., 2010; Lachance and Tishkoff, 2013).

In terms of economics, the greatest impact of genomics in livestock has been its application to breeding (i.e., genomic selection, GS). However, a large number of phenotyped (i.e., directly or through daughter/relative performances) and genotyped individuals are necessary to obtain accurate genomic breeding values (Goddard and Hayes, 2009). Breeding schemes combining traditional and genomic information have been proven to obtain good results in medium-scale breeds (Thomasen et al., 2014). However, considering the low number of individuals in small populations, only small gains from the use of genomic information can be expected (Pryce and Daetwyler, 2012). One possible solution is multi- or across-breed prediction, although its utility depends on various factors, including genetic distances among populations and the trait(s) considered (Lund et al., 2014). Such strategies have been successfully tested on relatively large dairy cattle breeds (Lund et al., 2011; Hozé et al., 2014), but are yet untested on small breeds. Given that application of GS in small breeds would require high-density genotyping, once again a specific cost–benefit analysis should be carefully considered. To face this issue, statistical methods that integrate genotyped and ungenotyped individuals could be adopted (Misztal et al., 2009). Another option, especially in populations where pedigree information is known, could be to genotype key ancestors at high-density and the rest of the population at low-medium density (Hozé et al., 2014). Imputation methods, also with a multi-breed reference population, can then be applied to obtain high genotyping accuracies for all animals (Berry et al., 2014).

### INNOVATIVE PHENOTYPES

Technological developments in agriculture have impacted not only the field of genomics, but also the collection of phenotypes. Animal phenotyping has mainly taken two directions: on one hand, the measurement of an increasingly large array of new phenotypes (Houle et al., 2010); on the other, the development of systems for automatic trait measurement and recording (e.g., Berry et al., 2012).

Genomic selection, in which trait measurement is limited to the reference population, contributed to put emphasis on the collection of novel phenotypes (e.g., Schöpke, 2014). For instance, milk quality traits now include not only total protein and fat content but also sub-components like lactoferrin and fatty acids (see RobustMilk, 2012). Mid- and near-infrared spectroscopy (MIR/NIR) allow quantitative evaluation of the composition of biological samples and have found wide application in dairy cattle breeding (e.g., De Marchi et al., 2014). Health-related traits represent yet another field of phenotypic investigation and include direct veterinary records, indirect measures of mastitis (e.g., milk electrical conductivity, milk mineral content) and female fertility (e.g., milk hormone assays, physical activity), and traits related to lameness or metabolic syndromes (Egger-Danner et al., 2014). Growing interest is being placed on behavioral traits like cow temperament (König et al., 2006) or feather pecking in laying hens (e.g., Biscarini et al., 2010).

Falling genotyping prices have left trait measurement as the major economically limiting factor in livestock selection schemes, thereby motivating active research in the (semi)automatic acquisition of phenotypes on a large scale. Automated milk-recording systems are becoming popular (e.g., Biscarini et al., 2012). The industry has been developing sensors to automatically measure many traits of direct or indirect interest. Pedometers for ambulatory activity indirectly measure fertility and lameness, and rumination tags monitor rumination time, which is related to metabolic activity and methane emissions (Soriani et al., 2012; Methagene, 2014). Image and video analysis can yield predictors of meat yield and quality (Pabiou et al., 2010) or body condition (Berry et al., 2012).

The combination of novel phenotypes and automatic trait recording is a powerful tool to improve both herd management and breeding schemes. Unfortunately, similar to GS (and conventional selection, for that matter), economies of scale are usually important in making the application of these technologies affordable and, therefore, innovative phenotyping is currently affecting mainly large commercial livestock populations. Nevertheless, this trend can still be a very promising development for smaller local breeds, inasmuch as the new technologies can be developed and perfected in larger populations, increasing their efficiency and decreasing costs so that can eventually help fill the gap toward optimized breeding and management practices in small breeds.

### BREEDING STRATEGIES

The small census size of local breeds does bring potential advantages. For example, implementation of OC selection requires decision-making to be centralized, which is impossible in large commercial populations where autonomy is widely dispersed and breeding organizations compete. In local breeds, fewer stakeholders are involved, so such coordination may be possible. Haile-Mariam et al. (2007) proposed a practical method to maximize genetic gain and minimize inbreeding by selection of dairy bulls on the genetic index of their progeny weighted by the cost of their expected inbreeding, a method that—due to its simplicity—could be promoted in local breeds. However, to date the OC method has been mainly analyzed in simplified populations with high selection intensities and rarely in multi stage selection schemes (Hinrichs and Meuwissen, 2011) and under the conditions encountered in local breeds (Gourdine et al., 2012; Gandini et al., 2014a,b).

**FIGURE 1:**
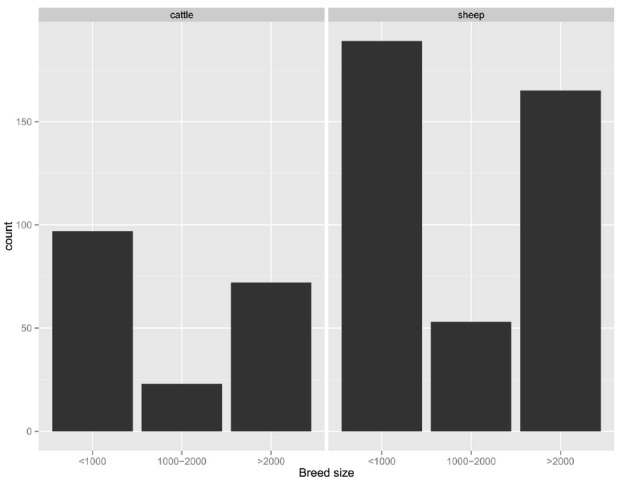
**Distribution of world cattle and sheep breeds according to the number of breeding females (data source DAD-IS: www.dad.fao.org, accessed on January 13, 2015): <1,000, 1,000–2,000, >2,000**.

Gourdine et al. (2012) simulated a breeding program aimed to improve meat quality in a local pig breed farmed in low-input systems with a given herd structure. OC selection at inbreeding rates around 0.001 per generation was shown to achieve reasonable gains. In dairy cattle populations with 500–6,000 females it has been shown that substantial genetic gain—about 50–70% of that achievable in large populations—can be obtained at an inbreeding rate per generation of about 0.001 (Gandini et al., 2014a). FAO (2013) advices selection for production to be implemented in breeds categorized as “vulnerable”; in cattle and sheep this translates to breeds with a number of breeding females between 1,000 and 2,000. Breeds with a larger breeding population are not considered at risk, while breeds with less than 1,000 breeding females are regarded as endangered: in this latter group of breeds selection programs are not advised. Figure 1 shows the number of cattle and sheep breeds registered in the FAO database DAD-IS^3^ under the vulnerable category, corresponding to 23 and 53 potential candidate breeds for selection programs in cattle and sheep, respectively.

**FIGURE 2:**
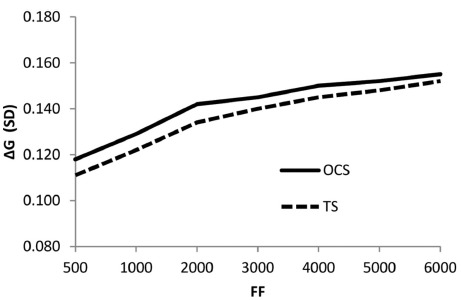
**Annual genetic gain (ΔG) (standard deviation units) with optimum contribution selection (OCS) and truncation selection (TS) in populations from 500 to 6,000 females (FF) with inbreeding control (Gandini et al., 2014b)**.

Figure 2 shows the genetic gain as a function of population size under a young bull scheme with OC selection, compared to truncation selection at the same rate of inbreeding of 0.001 per generation (Gandini et al., 2014a). Other studies have reported much greater potential advantages of OC selection, from 16 to 44% relative to truncation selection; these were due to the much higher selection intensity and index accuracy used in these simulations (Gandini et al., 2014a). Kosgey et al. (2006), based on an analysis of selection for small ruminants in the tropics, underline that the success of breeding programs is mainly determined by their compatibility with the farming conditions and the involvement of the farmers, and that simplicity and applicability of the systems should be a major criterion in designing the breeding scheme. Close cooperation with farmers will also facilitate adoption of complementary practices, such as niche marketing and exploitation of breeds’ environmental services (FAO, 2013), which may be as important as genetic improvement in achieving profitability.

When pedigree and performance recording is limited, as often it occurs in the local breed scenario, genetic improvement can be generated in a small fraction of the population, the nucleus (e.g., Roden, 1994) and then disseminated to the whole population, with or without the use of artificial insemination. Annual genetic gains ranging from a minimum of 0.073 SD/generation (100-female nucleus for a commercial population of 500 females) to a maximum of 0.138 SD/generation (400-female nucleus for a commercial population of 5,000 females) have been simulated in small ruminant populations (Gandini et al., 2014a). A limitation with some nucleus schemes can be the genetic lag occurring between the commercial population and the nucleus (Bichard, 1971).

## CONCLUSION

Small local breeds face many challenges in maintaining or increasing their population sizes in order to avoid extinction. Their inferior productivity (relative to larger breeds) is a common reason why many small breeds are small. In turn, their size inhibits small breeds from exploiting economies of scale afforded to large breeds in genetic selection. Nevertheless, some form of genetic improvement is almost necessary for small breeds to have any hope for long-term survival. Although small breeds may not be able to fully utilize technological advances such as GS and innovative phenotyping, they can benefit from the ongoing development and use of these technologies in large breeds. Advanced methods to optimize genetic response and maintenance of diversity may actually be more easily applied in breeds with small population sizes and fewer stakeholders. Application of a battery of genetic tools, along with close cooperation with breeders and utilization of other tools such as innovative product marketing (FAO, 2013) may allow small breeds to not only survive, but also thrive.

### Conflict of Interest Statement

The authors declare that the research was conducted in the absence of any commercial or financial relationships that could be construed as a potential conflict of interest.

## Footnotes

1 https://www.crv4all.nl/service/mrij

2 http://www.icbf.com/

3 www.dad.fao.org, accessed on January 13, 2015

## References

[B1] Albrechtsen A. Nielsen F. C. Nielsen R. (2010). Ascertainment biases in SNP chips affect measures of population divergence. Mol. Biol. Evol. 27, 2534–2547. 10.1093/molbev/msq148 20558595 PMC3107607

[B2] Avendaño S. Villanueva B. Woolliams J. A. (2003). Expected increases in genetic merit from using optimized contributions in two livestock populations of beef cattle and sheep. J. Anim. Sci. 81, 2964–2975. 14677851 10.2527/2003.81122964x

[B3] Berry D. P. Cromie A. R. McHugh N. Burke M. Pabiou T. MacCarthy J. (2012). “New traits for dairy cattle breeding,” in Proceedings 38th International Committee for Animal Recording (ICAR) Meeting, Cork, Ireland.

[B4] Berry D. P. McClure M. C. Mullen M. P. (2014). Within- and across-breed imputation of high-density genotypes in dairy and beef cattle from medium- and low-density genotypes. J. Anim. Breed. Genet. 13, 165–172. 10.1111/jbg.12067 24906026

[B5] Bichard M. (1971). Dissemination of genetic improvement through a livestock industry. Anim. Prod. 13, 401–411 10.1017/S0003356100010606

[B6] Biscarini F. Bovenhuis H. Van Der Poel J. Rodenburg T. B. Jungerius A. P. Van Arendonk J. A. M. (2010). Across-line SNP association study for direct and associative effects on feather damage in laying hens. Behav. Genet. 40, 715–727. 10.1007/s10519-010-9370-0 20496162

[B7] Biscarini F. Nicolazzi E. L. Stella A. the Prozoo Team. (2012). “Automated milk-recording systems: an experience in Italian dairy cattle farms,” in Proceedings of the Book of Abstracts of the 63rd Annual Meeting of the EAAP, 27–31 August 2012, Bratislava, Slovakia, 95.

[B8] Brisbane J. R. Gibson J. P. (1995). Balancing selection response and rate of inbreeding by including genetic relationship in selection decisions. Theor. Appl. Genet. 91, 421–431. 24169831 10.1007/BF00222969

[B9] De Marchi M. Toffanin V. Cassandro M. Penasa M. (2014). Mid-infrared spectroscopy as phenotyping tool for milk traits. J. Dairy Sci. 97, 1171–1186. 10.3168/jds.2013-6799 24440251

[B10] de Simoni Gouveia J. J. Barbosa da Silva M. V. G. Rezende Paiva S. Pinheiro de Oliveira S. M. (2014). Identification of selection signatures in livestock species. Genet. Mol. Biol. 37, 330–342. 10.1590/S1415-47572014000300004 25071397 PMC4094609

[B11] Egger-Danner C. Cole J. B. Pryce J. E. Gengler N. Heringstad B. Bradley A. (2014). Overview of new traits and phenotyping strategies in dairy cattle with a focus on functional traits. Animal 9, 191–207. 10.1017/S1751731114002614 25387784 PMC4299537

[B12] FAO. (2007). The Global Strategy for the Management of Farm Animal Genetic Resources and the Interlaken Declaration. Available at: ftp://ftp.fao.org/docrep/fao/meeting/014/j9556e.pdf (accessed October 10, 2014).

[B13] FAO. (2011). Guidelines: Molecular Genetic Characterization of Animal Genetic Resources. Available at: http://www.fao.org/docrep/014/i2413e/i2413e00.htm (accessed October 10, 2014).

[B14] FAO. (2013). In Vivo Conservation of Animal Genetic Resources. FAO Animal Production and Health Guidelines. No. 14. Rome: FAO.

[B15] Gandini G. Del Corvo M. Biscarini F. Stella A. (2014a). Genetic improvement of small ruminant local breeds with nucleus and inbreeding control: a simulation study. Small Rumin. Res. 120, 196–203 10.1016/j.smallrumres.2014.06.004

[B16] Gandini G. Stella A. Del Corvo M. Jansen G. B. (2014b). Selection with inbreeding control in simulated young bull schemes for local dairy cattle breeds. J. Dairy Sci. 97, 1790–1798. 10.3168/jds.2013-7184 24440254

[B17] Gandini G. Maltecca C. Pizzi F. Bagnato A. Rizzi R. (2007). Comparing local and commercial breeds on functional traits and profitability: the case of Reggiana dairy cattle. J. Dairy Sci. 90, 2004–2011. 10.3168/jds.2006-204 17369242

[B18] Goddard M. E. Hayes B. J. (2009). Mapping genes for complex traits in domestic animals and their use in breeding programmes. Nat. Rev. Genet. 10, 381–391. 10.1038/nrg2575 19448663

[B19] Gourdine J. L. Sorenson A. C. Rydhmer L. (2012). There is room for selection in a small local pig breed when using optimum contribution selection: a simulation study. J. Anim. Sci. 90, 76–84. 10.2527/jas.2011-3898 21841085

[B20] Grundy B., Caballero, A., Santiago, E., Hill W. G. (1994). A note on using biased parameter values and non-random mating to reduce rates of inbreeding in selection programmes. Anim. Prod. 59, 465–468 10.1017/S0003356100008011

[B21] Grundy B. Villanueva B. Woolliams J. A. (1998). Dynamic selection procedures for constrained inbreeding and their consequences for pedigree development. Genet. Res. 72, 159–168 10.1017/S0016672398003474

[B22] Haile-Mariam M. Bowman P. J. Goddard M. (2007). A practical approach for minimising inbreeding and maximising genetic gain in dairy cattle. Genet. Sel. Evol. 30, 369–389 10.1186/1297-9686-39-4-369 17612478 PMC2682817

[B23] Hinrichs D. Meuwissen T. H. E. (2011). Analyzing the effect of different approaches of penalized relationship in multi stage selection schemes. J. Anim. Sci. 89, 3426–3432. 10.2527/jas.2010-3621 21705631

[B24] Houle D. Govindaraju D. R. Omholt S. (2010). Phenomics: the next challenge. Nat. Rev. Genet. 11, 855–866. 10.1038/nrg2897 21085204

[B25] Hozé C. Fritz S. Phocas F. Boichard D. Ducrocq V. Croiseau P. (2014). Efficiency of multi-breed genomic selection for dairy cattle breeds with different sizes of reference population. J. Dairy Sci. 97, 3918–3929. 10.3168/jds.2013-7761 24704232

[B26] Huson H. J. Kim E. S. Godfrey R. W. Olson T. A. McClure M. C. Chase C. C. (2014). Genome-wide association study and ancestral origins of the slick-hair coat in tropically adapted cattle. Front. Genet. 5:101. 10.3389/fgene.2014.00101 24808908 PMC4010767

[B27] Joost S. Vuilleumier S. Jensen J. D. Schoville S. Leempoel K. Stucki S. (2013). Uncovering the genetic basis of adaptive change: on the intersection of landscape genomics and theoretical population genetics. Mol. Ecol. 22, 3659–3665. 10.1111/mec.12352 24003454

[B28] Koenig S. Simianer H. (2006). Approaches to the management of inbreeding and relationship in the German Holstein dairy cattle population. Livest. Sci. 103, 40–53 10.1016/j.livsci.2005.12.009

[B29] König S. Köhn F. Kuwan K. Simianer H. Gauly M. (2006). Use of repeated measures analysis for evaluation of genetic background of dairy cattle behavior in automatic milking systems. J. Dairy Sci. 89, 3636–3644 10.3168/jds.S0022-0302(06)72403-1 16899699

[B30] Kosgey I. S. Baker R. L. Udo H. M. J. Van Arendonk J. A. M. (2006). Successes and failures of small ruminant breeding programmes in the tropics: a review. Small Rumin. Res. 61, 13–28 10.1016/j.smallrumres.2005.01.003

[B31] Lachance J. Tishkoff S. A. (2013). SNP ascertainment bias in population genetic analyses: why it is important, and how to correct it. Bioessays 35, 780–786. 10.1002/bies.201300014 23836388 PMC3849385

[B32] Lenstra J. L. Groeneveld L. F. Eding H. Kantanen J. Williams J. L. Taberlet P. (2012). Molecular tools and analytical approaches for the characterization of farm animal genetic diversity. Anim. Genet. 43, 483–502. 10.1111/j.1365-2052.2011.02309.x 22497351

[B33] Lund M. S. de Roos A. P. V. de Vries A. G. Druet T. Ducrocq V. Fritz S. (2011). A common reference population from four European Holstein populations increases reliability of genomic predictions. Genet. Sel. Evol. 43, 43. 10.1186/1297-9686-43-43 22152008 PMC3292506

[B34] Lund M. S. Su G. Janss L. Guldbrandtsen B. Brondum R. F. (2014). Genomic evaluation of cattle in a multi-breed context. Livest. Sci. 166, 101–110 10.1016/j.livsci.2014.05.008

[B35] Manichaikul A. Mychaleckyj J. C. Rich S. S. Daly K. Sale M. Chen W. M. (2010). Robust relationship inference in genome-wide association studies. Bioinformatics 26, 2867–2873. 10.1093/bioinformatics/btq559 20926424 PMC3025716

[B36] Martín-Collado D. Díaz C. Mäki-Tanila A. Colinet F. Duclos D. Hiemstra S. J. (2013). The use of SWOT analysis to explore and prioritize conservation and development strategies for local cattle breeds. Animal 7, 885–894. 10.1017/S175173111200242X 23254176

[B37] Methagene. (2014). Large-scale Methane Measurements on Individual Ruminants for Genetic Evaluations. Available at: http://www.methagene.eu/ (accessed October 15, 2014).

[B38] Meuwissen T. H. E. (1997). Maximizing the response of selection with predefined rate of inbreeding. J. Anim. Sci. 75, 934–940. 9110204 10.2527/1997.754934x

[B39] Meuwissen T. H. E. Woolliams J. A. (1994). Effective size of livestock populations to prevent a decline in fitness. Theor. Appl. Genet. 89, 10119–11026. 10.1007/BF00224533 24178119

[B40] Misztal I. Legarra A. Aguilar I. (2009). Computing procedures for genetic evaluation including phenotypic, full pedigree, and genomic information. J. Dairy Sci. 92, 4648–4655. 10.3168/jds.2009-2064 19700728

[B41] Nicoloso L. Crepaldi P. Mazza R. Ajmone-Marsan P. Negrini R. (2013). Recent advance in DNA-based traceability and authentication of livestock meat PDO and PGI products. Recent Pat. Food Nutr. Agric. 5, 9–18. 10.2174/2212798411305010004 23305425

[B42] Ollivier L. Foulley J. (2005). Aggregate diversity: new approach combining within and between breed genetic diversity. Livest. Prod. Sci. 95, 247–254 10.1016/j.livprodsci.2005.01.005

[B43] Pabiou T. Fikse W. F. Cromie A. R. Kean M. G. Näsholm A. Berry D. P. (2010). Use of digital images to predict carcass cut yields in cattle. Livest. Sci. 137, 130–140 10.1016/j.livsci.2010.10.012

[B44] Pirola Y. Della Vedova G. Bonizzoni P. Stella A. Biscarini F. (2013). “Haplotype-based prediction of gene alleles using pedigrees and SNP genotypes,” in Proceedings of the International Conference on Bioinformatics, Computational Biology and Biomedical Informatics (BCB’13). New York, NY: ACM, 9 pages.

[B45] Pryce J. E. Daetwyler H. D. (2012). Designing dairy cattle breeding schemes under genomic selection: a review of international research. Anim. Prod. Sci. 52, 107–114 10.1071/AN11098

[B46] Purfield D. C. Berry D. P. McParland S. Bradley D. G. (2012). Runs of homozygosity and population history in cattle. BMC Genet. 13:70. 10.1186/1471-2156-13-70 22888858 PMC3502433

[B47] Randhawa I. A. S. Khatkar M. S. Campbell Thomson P. Raadsma H. W. (2014). Composite selection signals can localize the trait specific genomic regions in multi-breed populations of cattle and sheep. BMC Genet. 15:34. 10.1186/1471-2156-15-34 24636660 PMC4101850

[B48] RobustMilk. (2012). Innovative and Practical Breeding Tools for Improved Dairy Products from More Robust Dairy Cattle. Available at: http://www.robustmilk.eu/ (accessed October 15, 2014).

[B49] Roden J. A. (1994). Review of the theory of open nucleus breeding systems. Anim. Breed. Abstr. 62, 151–157.

[B50] Rothschild M. F. Plastow G. S. (2014). Applications of genomics to improve livestock in the developing world. Livest. Sci. 166, 76–83 10.1016/j.livsci.2014.03.020

[B51] Schöpke K. (2014). “*Assembling a Reference Population—From Genetic Architecture to New Phenotypes*,” in Proceedings of the 10th WCGALP, Vancouver.

[B52] Somavilla A. L. Sonstegard T. S. Higa R. H. Rosa A. N. Siqueira F. Silva L. O. C. (2014). A genome-wide scan for selection signatures in Nellore cattle. Anim. Genet. 45, 771–781. 10.1111/age.12210 25183526

[B53] Soriani N. Trevisi E. Calamari L. (2012). Relationships between rumination time, metabolic conditions, and health status in dairy cows during the transition period. J. Anim. Sci. 90, 4544–4554. 10.2527/jas.2011-5064 23255819

[B54] Stella A. Ajmone-Marsan P. Lazzari B. Boettcher P. (2010). Identification of selection signatures in cattle breeds selected for dairy production. Genetics 185, 1451–1461. 10.1534/genetics.110.116111 20479146 PMC2927769

[B55] Thomasen J. R. Egger-Danner C. Willam A. Guldbrandtsen B. Lund M. S. Sorensen A. C. (2014). Genomic selection strategies in a small dairy cattle population evaluated for genetic gain and profit. J. Dairy Sci. 97, 458–470. 10.3168/jds.2013-6599 24239076

[B56] Utsunomiya Y. T. Bomba L. B. Lucente G. Colli L. Negrini R. Lenstra J. A. (2014). Revisiting AFLP fingerprinting for an unbiased assessment of genetic structure and differentiation of taurine and zebu cattle. BMC Genet. 15:47. 10.1186/1471-2156-15-47 24739206 PMC4021504

[B57] VanRaden P. M. Olson K. M. Wiggans G. R. Cole J. B. Tooker M. E. (2011a). Genomic inbreeding and relationships among Holsteins, Jerseys, and Brown Swiss. J. Dairy Sci. 94, 5673–5682. 10.3168/jds.2011-4500 22032391

[B58] VanRaden P. M. Olson K. M. Null D. J. Hutchison J. L. (2011b). Harmful recessive effects on fertility detected by absence of homozygous haplotypes. J. Dairy Sci. 94, 6153–6161. 10.3168/jds.2011-4624 22118103

[B59] Villanueva B. Woolliams J. A. Simm G. (1994). Strategies for controlling rates of inbreeding in MOET nucleus schemes for beef cattle. Genet. Sel. Evol. 25, 517–535 10.1186/1297-9686-26-6-517

[B60] Wang C. Da Y. (2014). Quantitative genetics model as the unifying model for defining genomic relationship and inbreeding coefficient. PLoS ONE 9:e114484. 10.1371/journal.pone.0114484 25517971 PMC4269408

[B61] Wray N. R. Thompson R. (1990). Prediction of rates of inbreeding in selected populations. Genet. Res. 55, 41–54. 10.1017/S0016672300025180 2318415

